# Mental Training Affects Distribution of Limited Brain Resources 

**DOI:** 10.1371/journal.pbio.0050138

**Published:** 2007-05-08

**Authors:** Heleen A Slagter, Antoine Lutz, Lawrence L Greischar, Andrew D Francis, Sander Nieuwenhuis, James M Davis, Richard J Davidson

**Affiliations:** 1 Waisman Laboratory for Brain Imaging and Behavior and Department of Psychology, University of Wisconsin, Madison, Wisconsin, United States of America; 2 Department of Psychology, Leiden University, Leiden, The Netherlands; University of California Irvine, United States of America

## Abstract

The information processing capacity of the human mind is limited, as is evidenced by the so-called “attentional-blink” deficit: When two targets (T1 and T2) embedded in a rapid stream of events are presented in close temporal proximity, the second target is often not seen. This deficit is believed to result from competition between the two targets for limited attentional resources. Here we show, using performance in an attentional-blink task and scalp-recorded brain potentials, that meditation, or mental training, affects the distribution of limited brain resources. Three months of intensive mental training resulted in a smaller attentional blink and reduced brain-resource allocation to the first target, as reflected by a smaller T1-elicited P3b, a brain-potential index of resource allocation. Furthermore, those individuals that showed the largest decrease in brain-resource allocation to T1 generally showed the greatest reduction in attentional-blink size. These observations provide novel support for the view that the ability to accurately identify T2 depends upon the efficient deployment of resources to T1. The results also demonstrate that mental training can result in increased control over the distribution of limited brain resources. Our study supports the idea that plasticity in brain and mental function exists throughout life and illustrates the usefulness of systematic mental training in the study of the human mind.

## Author Summary

Meditation includes the mental training of attention, which involves the selection of goal-relevant information from the array of inputs that bombard our sensory systems. One of the major limitations of the attentional system concerns the ability to process two temporally close, task-relevant stimuli. When the second of two target stimuli is presented within a half second of the first one in a rapid sequence of events, it is often not detected. This so-called “attentional-blink” deficit is thought to result from competition between stimuli for limited attentional resources. We measured the effects of intense meditation on performance and scalp-recorded brain potentials in an attentional-blink task. We found that three months of intensive meditation reduced brain-resource allocation to the first target, enabling practitioners to more often detect the second target with no compromise in their ability to detect the first target. These findings demonstrate that meditative training can improve performance on a novel task that requires the trained attentional abilities.

## Abstract 3

Intensive training in Vipassana meditation enhances one's ability to allocate attention efficiently in order to detect visual targets accurately. Behavioral and event-related potential evidence for a causal link between behavioral training and brain plasticity in adults is shown.

## Introduction

A major limitation in human information processing arises from the time required to consciously identify and consolidate a visual stimulus in short-term memory [1]. This process can take more than a half-second before it is free for a second stimulus, as is revealed by the attentional-blink paradigm: If the second target stimulus (T2) of two target stimuli is presented within 500 ms of the first one (T1) in a rapid sequence of distracters, it is often not detected [2,3]. This deficit in the ability to process two temporally close, meaningful stimuli is believed to result from competition between the two targets for limited attentional resources [4]: When many attentional resources are devoted to the processing of T1, too few may be available for T2, rendering its representation vulnerable to distracter interference. Yet, the attentional blink does not reflect a general, immutable bottleneck, because most individuals are able to identify T2 on at least a portion of trials (e.g., [5]). This suggests that some control (which need not be voluntary) over the allocation of attentional resources is possible.

The current study examined whether intensive meditation can affect the distribution of limited attentional resources, as measured by performance in an attentional-blink task and scalp-recorded brain potentials. A major ingredient of meditation is mental training of attention. Such mental training is thought to produce lasting changes in brain and cognitive function, significantly affecting the way stimuli are processed and perceived. In line with this view, recent studies have reported cognitive and neural differences in attentional processing between expert meditators and novices [6,7]. However, to corroborate the idea that mental processes are flexible skills that can be trained through meditation, longitudinal data examining such changes over time within the same individuals are required. Previous research in non-practitioners has shown that the adult human brain is capable of plastic change in response to environmental stimulation (e.g., [8,9]) and that intensive training of an external task, such as a computer game, can improve attention skills, as reflected by enhanced performance on new cognitive tasks [10]. Yet, it is unclear at present whether purely mental training of certain attentional skills can benefit performance on novel tasks, which do not require meditation, but do call upon the trained skills.

Here we present a longitudinal study investigating effects of 3 mo of intensive Vipassana meditation on the distribution of limited attentional resources. In this common style of meditation, one starts by focusing or stabilizing concentration on an object such as the breath. Then one broadens one's focus, cultivating a non-reactive form of sensory awareness or “bare” attention. This form of attention is non-reactive in the sense that, ideally one does not become caught up in judgments and affective responses about sensory or mental stimuli. On the basis of previous findings in expert meditators [6,7], we hypothesized that 3 mo of intensive Vipassana meditation training would produce significant changes in attentional processing. More specifically, as this style of meditation cultivates non-reactive awareness, we predicted that after 3 mo of intensive practice, (1) attention would be captured less by T1, resulting in a smaller attentional blink for T2; and (2) this reduction in T1 capture would be reflected in a smaller T1-elicited P3b, a brain-potential index of resource allocation [11].

**Figure 1:**
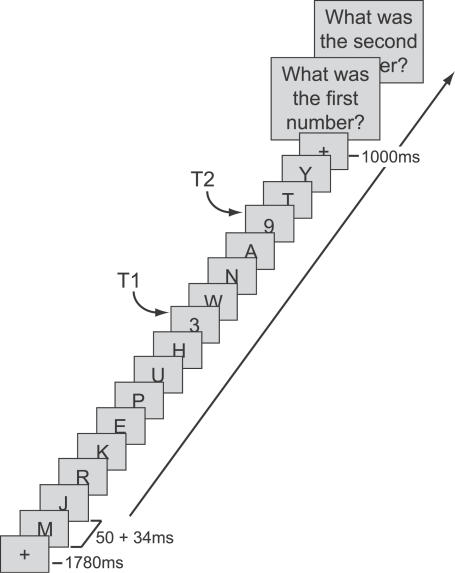
Attentional-Blink Task On every trial, between 15 and 19 items were presented at the center of the screen, preceded by a 1,780-ms fixation cross. Most of the items were letters, presented for 50 ms each and followed by a 34-ms blank. On T2-present trials, there were two target numbers (T1 and T2) among the items, which participants had to detect and report at the end of the trial. The temporal distance between T1 and T2 could be short (336 ms) or long (672 ms).

Data were collected from 17 participants at the beginning and end of a 3-mo meditation retreat during which they meditated for 10–12 h per day (practitioner group). Control data were collected from 23 participants interested in learning about meditation (novice group), who received a 1-h meditation class and were asked to meditate for 20 min daily for 1 w prior to each session. In each session, participants performed an attentional-blink task in which they had to identify two targets (both numbers) embedded in a rapid stream of distracter letters (Figure 1). T2 could follow T1 after either a short or a long interval, so that T2 could occur within (336 ms post T1) or outside (672 ms post T1) the attentional-blink time window. Participants were not engaged in formal meditation during task performance.

## Results

### Behavior

**Figure 2:**
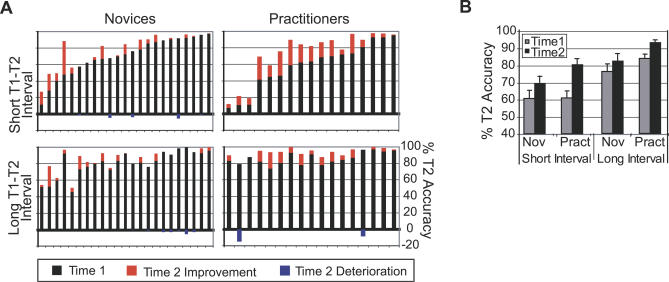
Effects of Intensive Mental Training on the Attentional Blink (A) T2 accuracy at time 1 (black bars) and the improvement (red bars) or deterioration (blue bars) in T2 accuracy at time 2, for each participant, separately for the short and long T1–T2 interval. (B) Average T2 accuracy (plus standard error) for each session, T1–T2 interval, and group (at-chance participants excluded). Note that both groups showed an attentional blink at time 1: lower T2 accuracy at short- compared to long-interval trials. Note further that, as predicted, the practitioner group (Pract) showed a significantly larger reduction in attentional-blink size over time than the novice group (Nov).

Our first prediction was that intensive mental training would reduce attentional capture by T1, as reflected by a smaller attentional blink for T2. The behavioral results showed that significantly more practitioners (17 out of 17) than novices (16 out of 23) showed higher T2 detection rates at the second session (time 2) when T2 followed T1 within the time window of the attentional blink (Figure 2A; Fisher exact test: *p* = 0.029). In line with previous reports (e.g., [5]), large variability between individuals of both groups was observed in attentional-blink size at the initial session (time 1). Three practitioners and one novice performed at, or near chance level at both time points (i.e., T2 accuracy around 14%). Regardless of whether or not these participants were included in the analysis, a significant reduction in attentional-blink magnitude over time was observed for the practitioner group compared to the novice group, as reflected by a three-way interaction between Interval, Group, and Session (Figure 2B; at-chance participants included: *F*(1,38) = 4.5, *p* = 0.040; at-chance participants excluded: *F*(1,34) = 4.3, *p* = 0.045). All two-way interaction terms and main effects shown in Figure 2B were significant (Table 1). Post hoc analyses confirmed that only the practitioner group showed a significantly smaller attentional blink at time 2 (interaction Interval by Time; Practitioners: *F*(1,16) = 16.1, *p* = 0.001; Novices: *F*(1,22) = 1.12, *p* > 0.05). In addition, the mental training-related improvement in T2 accuracy was selective to the time window of the attentional blink (interaction Group by Time; short-interval trials: *F*(1,38) = 7.4, *p* = 0.010; long-interval trials: *F*(1,38) = 0.7, *p* = 0.41).

**Table 1:**
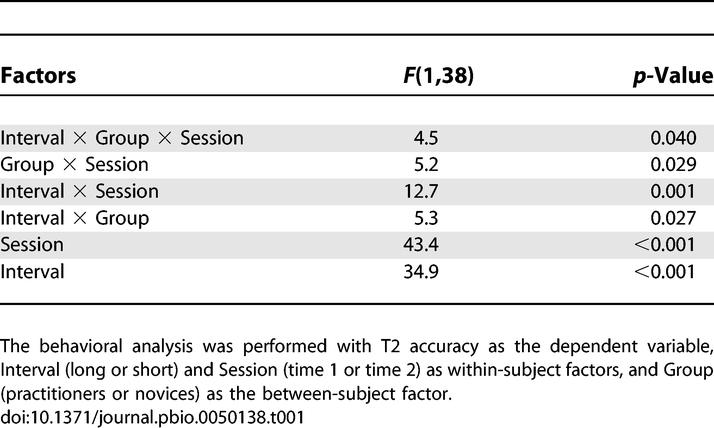
Behavioral Results (Repeated-Measures ANOVA)

It is possible that there was more room for improvement in T2 accuracy in those participants with lower T2 accuracy scores at time 1 and, therefore, that inter-individual differences in task performance at time 1 critically influenced the observed reduction in attentional-blink size over time in the practitioners. However, this possibility was excluded by the results of an analysis of covariance (ANCOVA) in which we controlled for T2 accuracy at time 1 by entering this measure as a covariate in the analysis. The difference in T2 accuracy at time 2 versus time 1 was used as the dependent variable and Group was used as a fixed factor. Importantly, the reduction in attentional-blink size over time was still significantly greater in the practitioner versus control group (*F*(1,37) = 5.1, *p* = 0.029), arguing against the possibility of differential room for improvement over time between groups.

One interesting observation was that although only the practitioners showed a smaller attentional blink at time 2, T2 accuracy generally improved slightly over time: both groups had significantly higher T2 accuracy scores at time 2 in both short-interval trials (novices: *t* = 2.9, *p* = 0.008; practitioners: *t* = 6.4, *p* < 0.001) and long-interval trials (novices: *t* = 2.8, *p* = 0.010; practitioners: *t* = 2.9, *p* = 0.011). T1 accuracy also generally improved slightly over time (*F*(1,38) = 8.1, *p* = 0.007). It is possible that with practice, the target stimuli became easier to detect as a result of perceptual learning [12] or that participants became better at predicting the timing of the target stimuli in the stream. Note, however, that such general practice effects cannot explain the observed selective improvement in T2 accuracy in short-interval trials in the practitioner group.

T1 accuracy was not significantly different between groups and, as mentioned above, improved slightly over time, indicating that improved T2 detection did not impair T1 detection. At time 1, the practitioners and novices accurately identified T1 on 78% and 79% of the short-interval trials, respectively, and on 88% and 88% of the long-interval trials, respectively. At time 2, the practitioners and novices accurately identified T1 on 83% and 82% of the short-interval trials, respectively, and on 91% and 91% of the long-interval trials, respectively. The design also included T2-absent trials in which only T1 was presented and T2 was replaced by a blank (see Materials and Methods). Average correct report of T2 absence showed no main effect of Session or any significant interactions, including Group, and will not be discussed further.

### Event-Related Potentials

The behavioral results showed that intensive mental training was associated with a significant reduction in attentional-blink size. We predicted that this reduction in attentional-blink size would be associated with a reduction in brain-resource allocation to T1, as reflected by a smaller T1-elicited P3b. The change in T1-elicited P3b over time was assessed in the 350–650-ms window for short-interval trials in which both targets were correctly identified (i.e., no-blink trials) and for trials in which only T1 was correctly identified (i.e., blink trials). For each sample and channel, voltage values in blink and no-blink trials were submitted to a repeated-measures analysis of variance (ANOVA) with T2 accuracy (blink or no-blink) and Session (time1 or time2) as within-subject factors and Group (practitioners or novices) as a between-subjects factor. Ten practitioners and 12 controls had enough artifact-free blink and no-blink electroencephalogram (EEG) trials (*n* > 15) at both time points to be included in this analysis. In line with the overall group behavioral findings, the reduction in blink size was significantly larger for the practitioner subgroup than for the novice subgroup (Interval × Group × Session interaction: *F*(1,20) = 5.4, *p* = 0.030; T2 accuracy in short-interval trials for the practitioners: 80% [time 2] vs. 61% [time 1], and the novices: 69% [time 2] vs. 60% [time1]). The average number of trials included in the event-related potential analysis was Novices: 73 (time 1) and 99 (time 2) no-blink trials, and 45 (time 1) and 40 (time 2) blink trials; Practitioners: 81 (time 1) and 121 (time 2) no-blink trials, and 51 (time 1) and 28 (time 2) blink trials.

**Figure 3:**
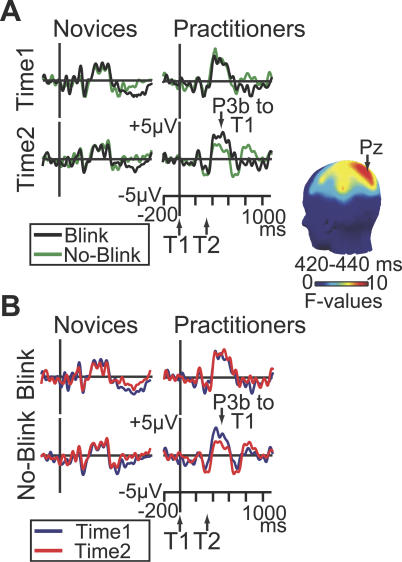
Effects of Intensive Mental Training on Brain-Resource Allocation to T1 (A) Brain potentials from electrode Pz, time-locked to T1 onset on short-interval trials as a function of T2 accuracy (no-blink vs. blink), session, and group. These data show that, as hypothesized, the practitioners showed a significantly greater reduction in T1-elicited P3b amplitude than the novices in no-blink versus blink trials at time 2 versus time 1. The scalp map shows electrode sites where this three-way interaction was significant between 420 and 440 ms. (B) Brain potentials from electrode Pz, time-locked to T1 onset on short-interval trials as a function of session, T2 accuracy, and group. This figure panel illustrates that intensive mental training was associated with a selective reduction in T1-elicited P3b amplitude in no-blink trials in the practitioner group.

In line with our prediction, intensive mental training was associated with a reduction in T1-elicited P3b amplitude over time in no-blink versus blink trials (Figure 3A). Significant Group × T2 accuracy × Session interaction effects were observed for the early phase of the P3b between 394–450 ms (largest at electrode Pz: *F*(1,20): 4.4–9.7, *p-*values < 0.05) and for its later phase between 488–551 ms post T1-onset (largest at CP3: *F*(1,20): 7.8–27.5, *p-*values < 0.05). Additional analyses revealed that both effects reflected a selective reduction across sessions of T1-elicited P3b amplitude in no-blink versus blink trials in practitioners (Figure 3B; early phase at Pz: *F*max(1,9) = 8.7, *p* = 0.016; late phase at CP3: *F*max(1,9) = 40.4, *p* = 0.0001). No such reduction in P3b amplitude was observed for novices (early phase: all *F-*values for *F*(1,11) < 1; late phase: all *F-*values for *F*(1,11) < 4.6).

**Figure 4:**
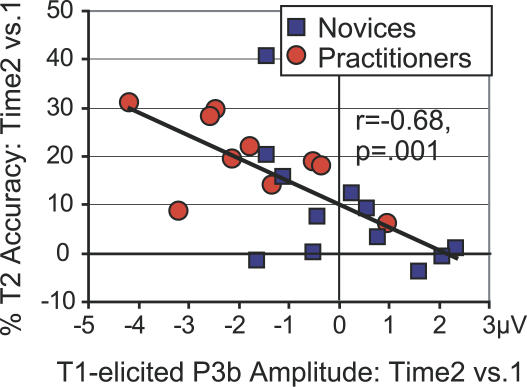
The Ability to Accurately Identify T2 Depends upon the Efficient Processing of T1 Relationship between the change in attentional-blink magnitude over time and the corresponding change in T1-elicited P3b amplitude (for no-blink trials). Note that those individuals that showed the largest decrease in T1-elicited P3b amplitude over time generally showed the largest increase in T2 accuracy over time.

As mentioned above, the attentional blink is thought to result from suboptimal sharing of limited attentional resources: When many resources are devoted to T1 processing, T2 is more likely to be missed [4,13]. We hypothesized, therefore, that the magnitude of decrease in T1-elicited P3b amplitude would be predictive of the magnitude of decrease in attentional-blink size. Indeed, correlation analyses revealed that those individuals—practitioner or novice—that showed the largest decrease in brain-resource allocation to T1 over time generally showed the greatest improvement in detecting T2: a reliable, negative cross-subject correlation was observed between the increase over time in T2 accuracy and the corresponding change in T1-elicited P3b amplitude on no-blink trials for both the early phase (Figure 4; *r* = −0.68, *p* = 0.001; data from Pz) and the late phase (*r* = −0.46, *p* = 0.030; data from CP3) of the P3b. Note, however, that as predicted, only the practitioners could exploit this resource-sharing mechanism: the P3b amplitude reduction to T1 at the time of the second recording was present only for this group and not for the novices (see above; see Figure 3A).

No significant Group × T2 accuracy × Session interaction effects were observed outside of the T1-elicited P3b window, including the time window of the P3b elicited by T2 (all *p-*values > 0.05). Previous brain-potential studies have reported that when T2 is not seen, the P3b to T2 is largely or completely suppressed [14,15]. Replicating these prior findings, we found greater positivity in no-blink versus blink trials in the time window of the T2-elicited P3b (800–1,000 ms), as reflected by significant main effects of T2 accuracy at several dorsal posterior electrode sites, including Pz (Practitioners: *F*(1,9): 5.1–9.9; Novices: *F*(1,11): 5.9–36.5; all *p-*values < 0.05).

**Figure 5:**
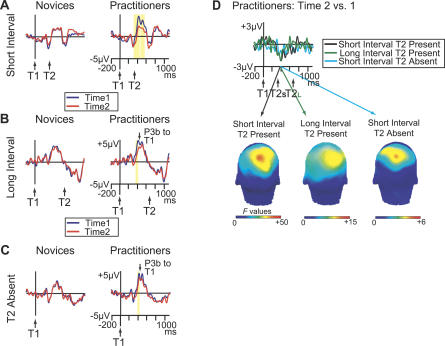
Mental Training Selectively Reduced Early T1-Evoked P3b Amplitude (A–C) Brain potentials from electrode Pz, time-locked to T1 onset as a function of Session (time 1 or time 2) and Group (novices or practitioners), shown separately for (A) short-interval T2-present no-blink trials, (B) long-interval T2-present no-blink trials, and (C) short-interval T2-absent trials. Yellow bars indicate the time windows in which the reduction in brain potential over time was significant in the time window of the T1-elicited P3b (*p* < 0.05). (D) The T1-locked change in brain potentials over time in practitioners from electrode Pz, shown separately for short-interval T2-present no-blink trials, long-interval T2-present no-blink trials, and short-interval T2 absent trials. The scalp maps show electrode sites where the change in T1-evoked P3b over time was significant between 380 and 400 ms, separately for each trial type. These data confirm that intensive mental training was associated with a selective reduction in T1-evoked P3b amplitude around 400 ms.

As effects of intensive mental training on scalp-recorded brain activity appeared specific to no-blink trials (see Figure 3B), we ran another analysis that included all participants that had enough no-blink trials (*n* > 15) at both time points (14 practitioners and 20 novices). This repeated-measures ANOVA using the within-subjects factor Session (time1 or time2) and the between-subjects factor Group (novices or practitioners) replicated the above described reductions in T1-elicited P3b amplitude over time in the practitioner group (see Figure 5A), as reflected by significant Session × Group interaction effects (early phase[316–445 ms] at Pz: *F*(1,33): 4.5–26.6, *p-*values < 0.05; late phase [477–563 ms] at CP3: *F*(1,33): 4.5–9.9, *p-*values < 0.05). Importantly, the correlation between the reduction in T1-elicited P3b amplitude and the increase in T2 accuracy over time also remained significant using this larger sample of participants (early phase: *r* = −0.42, *p* = 0.014; late phase: *r* = −0.36, *p* = 0.036), illustrating the robustness of this finding.

It is important to note that the scalp-recorded brain responses to T1 and T2 overlapped in time. Previous brain-potential studies have shown that while early visual processing of T2 is not affected by T2 visibility, T2 processing in no-blink and blink trials may differ as early as 170 ms post T2 [16]. It is therefore possible that the reduction in posterior positivity during the later part of the T1-evoked P3b observed in short-interval trials (i.e., 488–551 ms post-T1 or 152–215 ms post-T2) was due to differential T2 processing rather than differential T1 processing. To examine this possibility, we ran two additional analyses, focusing on trials in which the T1-elicited P3b was not confounded by neural activity associated with T2. The first analysis focused on long-interval T2-present no-blink trials, in which T2 followed T1 after 672 ms, i.e., after the T1-evoked P3b occurred. The second analysis focused on short-interval T2-absent trials in which no T2 was presented. If for these trial types we observed a similar reduction in scalp-recorded brain activity in the time window of the T1-elicited P3b as we did for short-interval T2-present trials, this would strengthen the conclusion that meditation training changed the neural processing of T1. All participants had enough trials to be included in the two additional analyses. The average number of trials included in the long-interval T2-present analysis was Novices: 45 (time 1) and 52 (time 2) trials; and Practitioners: 48 (time 1) and 53 (time 2) trials. The average number of trials included in the T2-absent analysis was Novices: 46 (time 1) and 49 (time 2) trials; and Practitioners: 46 (time 1) and 45 (time 2) trials.

Of central importance, in both long-interval T2-present trials (Figure 5B) and in T2-absent trials (Figure 5C), we observed a reduction in T1-elicited P3b amplitude (around 400 ms) for the practitioners only (long-interval T2-present trials at Pz [370–413 ms]: *F*(1,16): 4.7–8.3, *p-*values < 0.05; T2-absent trials at Pz [398–410 ms]: *F*(1,16): 4.57–5.31, *p-*values < 0.05). This reduction in early T1-elicited P3b was highly similar both in time course and scalp topography to the reduction in early T1-elicited P3b that we observed for short-interval T2-present no-blink trials, albeit less pronounced, in particular for T2-absent trials (Figure 5D). Similar effects were not observed for the novices (all *p-*values > 0.05). For long-interval T2-present trials, the selective reduction of T1-elicited P3b amplitude for the practitioners was expressed in a significant interaction between Group and Session (367 and 402 ms; largest at electrode P3: *F*(1,37): 4.2–6.6, *p-*values < 0.05). For T2-absent trials, the interaction did not reach significance (*p-*values > 0.05; *p*(min) = 0.12) (but see next paragraph). Interestingly, in contrast to short-interval T2-present trials, no mental training-related reduction in late T1-elicited P3b was observed in long-interval T2-present trials or in T2-absent trials. This indicates that intensive mental training may have reduced T1 capture, as well as affected early T2-related processes.

The short-interval T2-present trial analysis showed that the effects of intensive mental training on scalp-recorded brain potentials were selective to no-blink trials: only in no-blink trials, but not in blink trials, a reduction in brain activity in the T1-elicited P3b time window was observed (Figure 3B). The long-interval T2-present trial analysis and the T2-absent trial analysis may not have been as sensitive to detecting mental training-related effects as the short-interval T2-present trial analysis, because both these trial types included a mixture of “blink” and “no-blink” trials. As to long-interval T2-present trials: the longer interval between the two targets conceivably allowed for T2 detection even in those trials in which many resources were devoted to T1 processing and that would have resulted in an attentional blink had the T1–T2 interval been short. Long-interval no-blink T2-present trials thus include both “blink” and “no-blink” trials. A similar argument can be made for T2-absent trials: when a participant indicated that s/he did not see a second target in a T2-absent trial, this could have been because s/he just did not see a second target (a “blink” trial) or because s/he was absolutely certain that no T2 had been present in the stream (a “no-blink” trial). T2-absent trials can thus also be said to include a mixture of “blink” and “no-blink” trials. As the short-interval T2-present trial analysis showed that mental training-related effects were specific to no-blink trials, the additional long-interval T2-present and T2-absent trial analyses may therefore not have been as sensitive to detecting mental training-related changes over time. This argument receives critical support from an extra analysis examining whether the decrease in brain activity in the time window of the T1-elicited P3b in short-interval T2-present trials was smaller when averaging across no-blink and blink trials. This analysis showed that the mean decrease in T1-elicited P3b amplitude over time was significantly smaller when averaging across blink and no-blink trials compared to when averaging across no-blink trials alone: 1.24 versus 1.70 μV (*p* = 0.015; early phase P3b as measured at Pz) and 1.05 versus 1.60 μV (*p* = 0.008; late phase P3b). These findings indicate that the observed decreases in T1-elicited P3b in long-interval T2-present trials and in T2-absent trials likely were less pronounced, because both trial types include a mixture of blink and no-blink trials.

## Discussion

This study examined whether intensive mental training can affect one of the major capacity limits of information processing in the brain: the brain's limited ability to process two temporally close meaningful items. Using performance in an attentional-blink task and scalp-recorded brain potentials, we found, as predicted, that 3 mo of intensive mental training resulted in a smaller attentional blink and reduced brain-resource allocation to the first target, as reflected by a smaller T1-elicited P3b. Of central importance, those individuals that showed the largest decrease in brain-resource allocation to T1 generally showed the greatest reduction in attentional-blink size. These novel observations indicate that the ability to accurately identify T2 depends upon the efficient deployment of resources to T1 and provide direct support for the view that the attentional blink results from suboptimal resource sharing [5,13,15,16]. Importantly, they demonstrate that through mental training, increased control over the distribution of limited brain resources may be possible.

Because participants did not engage in formal meditation during task performance, the observed reduction in T1 capture after 3 mo of intensive meditation suggests that purely mental training of certain attention skills can influence performance on a novel task that calls upon those skills. Green and Bavelier [10] reported that intensive action video-game playing can improve attention skills, as reflected by enhanced performance on new cognitive tasks, including the attentional-blink task. Here, we show that improvements in performance of a novel, external task may also be achieved by pure mental training. As such, our findings extend previous research showing that the adult human brain is capable of plastic change in response to environmental stimulation (e.g., [8,9,10]). Note that it is unlikely that motivational differences between groups can explain our findings, because previous work has shown that motivating participants to do well on an attentional-blink task by paying them according to their performance does not affect the magnitude of the attentional blink [17]. In addition, the current findings corroborate previous findings in expert meditators [6,7] by showing longitudinally, within subjects, that attention processes are flexible skills, which can be enhanced through mental training. The observed reduction in T1 capture after 3 mo of intensive Vipassana meditation training confirms first-person reports that this style of meditation affects attentional processes and can significantly affect the way stimuli are processed and perceived. Future longitudinal studies are needed to examine how long effects of mental training on attention may persist and whether even shorter-term training may demonstrably benefit various attentional skills.

Although they differ in the specific mechanisms, cognitive accounts of the attentional blink have generally held that there is a capacity-limited stage in stimulus processing and that competition between different stimuli for limited processing resources underlies the attentional-blink deficit (e.g., [18–21]). In line with this idea, several recent brain-potential studies have shown that the ability to accurately identify T2 is related to the latency and/or amplitude of the T1-elicited P3b [5,13,16,22]. A delayed or larger T1-evoked P3b was observed in trials in which T2 was missed versus seen [16] and in individuals exhibiting a relatively large attentional blink [5,13]. In addition, the amplitude of the T1-evoked P3b has recently been shown to be dependent on T1 probability and T1 cue validity [22]. Together, these findings indicate that variability in the duration or difficulty of the T1 task (as indexed by T1-elicited P3b amplitude) may affect the severity of the competition between T1 and T2. In the current study, the magnitude of decrease in T1-elicited P3b amplitude over time in no-blink trials predicted the magnitude of decrease in attentional-blink size over time. This observation provides important additional support for the idea that the ability to accurately identify two temporally close, meaningful items depends upon the efficient deployment of resources to the first item [4,13]. Beyond this, the current study indicates that through mental training, people may gain some control (which need not be voluntary) over the amount of attentional resources devoted to the processing of the first item.

Vipassana meditation allegedly reduces ongoing mental noise in the brain, enabling the practitioner to remain in the present moment. Three months of intensive training in this style of meditation may therefore have decreased mental capture by any stimulus, i.e., distracters and targets alike [5], resulting in reduced distracter interference. Although we cannot fully exclude the possibility that reduced distracter interference may have contributed to our findings, mental training-related effects were not observed outside of the time window of the T1-elicited P3b, including the time window of the T2-elicited P3b. This observation supports the idea that intensive mental training selectively reduced brain-resource allocation to T1. Intensive mental training may, however, have affected relatively early T2-related processes; In trials in which the T1-elicited P3b was not confounded by neural activity associated with T2 (i.e., long-interval trials and T2-absent trials), a mental training-related reduction in posterior positivity was only observed for the early phase of the T1-elicited P3b (around 400 ms post-T1), but—in contrast to short-interval T2-present trials—not for its later phase (around 500 ms post-T1). The timing of this later effect (i.e., 152–215 ms post-T2) concurs with effects observed in a recent brain-potential study [16]. This study elegantly showed that brain events occurring as early as 170 ms post-T2 were affected by the conscious perception of T2. Three months of intensive mental training may therefore not only have reduced T1 capture, but may have also influenced relatively early T2-elicited processes. The current findings allow us to speculate on candidate brain structures that intensive Vipassana meditation training may affect. Previous neuroimaging studies have implicated a network of frontal, parietal, and temporal brain areas in the generation of the scalp-recorded P3b [23]. Activation of a similar network of brain areas has been associated with conscious target processing in the attentional-blink task [24]. Three months of intensive mental training may thus have affected the recruitment of this distributed neural network.

In summary, the results presented here are consistent with the idea that the ability to accurately identify T2 depends upon the efficient processing of T1. They furthermore demonstrate that, through mental training, increased control over the allocation of limited processing resources may be possible. Our study corroborates the idea that plasticity in brain and mental function exists throughout life, and illustrates the usefulness of systematic mental training in the study of the human mind.

## Materials and Methods

### Participants.

Seventeen practitioners (seven male; median age, 41 y, range 22–64 y; median education, 18 y) were recruited prior to the start of a 3-mo meditation retreat at the Insight Meditation Society in Barre, Massachusetts. Twenty-three matched controls (nine male; median age, 41 y, range 20–62 y; median education, 17 y) with no prior meditation experience were recruited via advertisements in local newspapers directed at individuals interested in learning about meditation. The participants had no history of mental or neurological illness, and gave informed consent to participate. Participants were trained in Vipassana meditation, which cultivates concentration and “bare” attention (see Introduction). Through this style of training, one allegedly is able to be more finely attentive to experience from moment to moment and gain insight into one's habits and assumptions about identity and emotions. Practitioners were also trained in “metta,” a loving kindness and compassion meditation.

The practitioners self-selected for the meditation group and all had prior experience with meditation. Practitioners differed greatly in the style(s) of meditation previously practiced (e.g., Vipassana, open presence, mantra, or yoga meditation), in the traditions of the learned meditation (e.g., Zen, Theravada, or Tibetan), and in the amount of prior meditation experience. No relationship was observed between prior meditation experience (i.e., number of days in a retreat prior to our study) and attentional-blink task performance at time 1. Prior meditation experience also did not interact with the meditation intervention, as there was no significant relationship between prior meditation experience and the change over time in attentional-blink task performance. The absence of an association between the amount of prior meditation training and our study results may be due to the fact that there was great variation across the practitioners in the styles and traditions of the previously learned meditation. Longitudinal research examining and comparing the effects of different styles of meditation on brain and mental function and the duration of such effects is needed.

### Stimuli and task.

Stimuli were presented in black on a gray (40 cd/m^2^) background at the center of a computer screen. Each trial started with a 1,780-ms fixation cross (0.5° × 0.5°), followed by a rapid serial stream of 15 or 19 letters (0.8° × 0.8°) (Figure 1). Each letter was randomly drawn (without replacement) from the alphabet (except B, I, O, Q, and S) and presented for 50 ms, followed by a 34-ms blank. On each trial, one or two letters were replaced with a number, randomly drawn (without replacement) from the set 2–9. When only one letter was replaced by a number, a second letter was replaced with a blank screen (T2-absent trials). The temporal distance between the first (T1) and second (T2) number (or the blank screen) could be short (336 ms) or long (672 ms). T2 and the blank screen were presented at temporal position 3–5 from the end of the stream. To prevent the saliency of T2-absent trials, each distracter could be replaced by an empty screen with a 20% probability, except those surrounding T1 and T2 and the last distracter in the sequence (cf. [25]).

Participants were informed that there could be one or two numbers in the letter stream, and, 1,000 ms after the stream ended, were asked to report these numbers by typing the numbers in order on a keyboard. Participants were instructed to guess T2 if they thought that T2 had been presented, but were not entirely sure about its identity. If they were absolutely sure that no T2 was presented, they entered zero for this number. A new trial began 200 ms after the second response. After a short practice block, participants performed four blocks of 102 trials each, consisting of 192 short-interval/T2-present trials, 72 long-interval/T2-present trials, 72 short-interval/T2-absent trials and 72 long-interval/T2-absent trials, all intermixed within blocks.

### Procedure.

In each session, after practicing the task first for 34 trials, participants performed four blocks of 102 trials of the attentional-blink task while their EEG was recorded. EEG was recorded at 512 Hz from 64 Ag–AgCl electrodes using the Active-Two system (BioSemi, http://www.biosemi.com). Additional electrodes recorded the right and left mastoid process and the electrooculogram.

### Behavioral data: Analyses.

T1 and T2 accuracy data were submitted to separate repeated-measures ANOVAs with Interval (short or long) and Session (time 1 or time 2) as within-subject factors, and Group (novices or practitioners) as a between-subject factor. T2 accuracy was based only on those trials in which T1 was correctly reported.

### EEG data: Analyses.

EEGLAB [26] and Matlab (Mathworks, http://www.mathworks.com) were used for off-line EEG data processing. Due to technical problems, the EEG data of two novices could not be analyzed. Data of the remaining participants were high-pass filtered (1 Hz), re-referenced to the average of both mastoids, and cleaned of large movement-related artifacts. Independent component analysis was then used to remove ocular artifacts [27]. For each condition of interest, trials were epoched in synchrony with T1 onset, and baseline-corrected (200 ms preceding T1). Trials with remaining artifacts (exceeding ±80 μV) were removed. The remaining trials were low-pass filtered (20 Hz) and averaged. The change in T1-elicited P3b over time was assessed in the 350–650-ms window. A significance criterion of *p* < 0.05 for at least 16 consecutive samples (31 ms) on at least four adjacent electrodes was used.

## Abbreviations

ANOVA: analysis of variance
EEG: electroencephalogram
T1: first target
T2: second target
